# Parental socioeconomic status is linked to cortical microstructure and language abilities in children and adolescents

**DOI:** 10.1016/j.dcn.2022.101132

**Published:** 2022-07-04

**Authors:** Linn B. Norbom, Jamie Hanson, Dennis van der Meer, Lia Ferschmann, Espen Røysamb, Tilmann von Soest, Ole A. Andreassen, Ingrid Agartz, Lars T. Westlye, Christian K. Tamnes

**Affiliations:** a PROMENTA Research Center, Department of Psychology, University of Oslo, Norway; b NORMENT, Institute of Clinical Medicine, University of Oslo, Norway; c Department of Psychiatric Research, Diakonhjemmet Hospital, Oslo, Norway; d Learning Research and Development Center University of Pittsburgh, USA; e Department of Psychology, University of Pittsburgh, USA; f School of Mental Health and Neuroscience, Faculty of Health, Medicine and Life Sciences, Maastricht University, the Netherlands; g K.G Jebsen Center for Neurodevelopmental Disorders, University of Oslo, Norway; h NORMENT, Division of Mental Health and Addiction, Oslo University Hospital & Institute of Clinical Medicine, University of Oslo, Norway; i Department of Psychology, University of Oslo, Norway; j Norwegian Institute of Public Health, Norway; k Centre for Psychiatry Research, Department of Clinical Neuroscience, Karolinska Institutet & Stockholm Health Care Services, Stockholm Region, Stockholm, Sweden

**Keywords:** Structural cortical development, Parental socioeconomic status, T1w/T2w-ratio, Childhood, Adolescence

## Abstract

Gradients in parental socioeconomic status (SES) are closely linked to important life outcomes in children and adolescents, such as cognitive abilities, school achievement, and mental health. Parental SES may also influence brain development, with several magnetic resonance imaging (MRI) studies reporting associations with youth brain morphometry. However, MRI signal intensity metrics have not been assessed, but could offer a microstructural correlate, thereby increasing our understanding of SES influences on neurobiology. We computed a parental SES score from family income, parental education and parental occupation, and assessed relations with cortical microstructure as measured by T1w/T2w ratio (n = 504, age = 3–21 years). We found negative age-stabile relations between parental SES and T1w/T2w ratio, indicating that youths from lower SES families have higher ratio in widespread frontal, temporal, medial parietal and occipital regions, possibly indicating a more developed cortex. Effect sizes were small, but larger than for conventional morphometric properties i.e. cortical surface area and thickness, which were not significantly associated with parental SES. Youths from lower SES families had poorer language related abilities, but microstructural differences did not mediate these relations. T1w/T2w ratio appears to be a sensitive imaging marker for further exploring the association between parental SES and child brain development.

## Highlights

• Composite and sub-factor scores of parental socioeconomic status.
• Youth cortical microstructure measured by T1w/T2w ratio (n = 504, age = 3–21 years).
• Widespread negative relations with socioeconomic status and regional T1w/T2w ratio.
• Youths from families with lower socioeconomic status had poorer language abilities.

## 1 Introduction

Childhood and adolescence are central periods for brain development, encompassing maturational processes characterized by re-organization and optimization (Jernigan et al., 2016, Lebel and Deoni, 2018, Norbom et al., 2021). These same periods, and adolescence in particular, also mark the onset for most mental disorders (Kessler et al., 2005, Steinhausen and Jakobsen, 2019). It is therefore vital to study environmental factors and experiences that, in complex interplay with genetic factors, influence brain development (Johnson et al., 2016). Socioeconomic status (SES) is a central environmental factor, though certainly showing high heritability (Ørstavik et al., 2014, Tambs et al., 2012), and predictor of important life outcomes such as intelligence, academic achievement, and mental health (Letourneau et al., 2013, Reiss, 2013, Sirin, 2005, von Stumm and Plomin, 2015, Zhang et al., 2020). Examining how SES is related to the developing brain in childhood and adolescence is therefore of importance for probing the underlying mechanisms of these effects.

SES is often quantified by some form of income metric, as well as education, and occupation, which are moderately correlated with each other (Farah, 2017). Low parental SES is associated with a broad array of negative outcomes for children and adolescents, including lower intelligence, a discrepancy that widens across childhood (Duyme et al., 1999, von Stumm and Plomin, 2015), lower school performance (Sirin, 2005), and poorer cognitive functioning (Zhang et al., 2020), including language and self-regulatory abilities (Palacios-Barrios and Hanson, 2019, Schwab and Lew-Williams, 2016). Moreover, youths in families with lower SES are 2–3 times more likely to suffer from mental health problems than their peers with higher SES (Letourneau et al., 2013, Reiss, 2013). Although the associations between parental SES and youth cognition and psychopathology are well established, the neurobiology relaying these relationships remains poorly understood.

Although SES has historically been viewed as a nuisance variable in magnetic resonance imaging (MRI) studies (Farah, 2017), a growing literature reports associations between parental SES and brain structure and function of children and adolescents (Buckley et al., 2019, Farah, 2017, Johnson et al., 2016). Findings include positive relations with amygdala (Hanson et al., 2015, Machlin et al., 2020, McDermott et al., 2019, Merz et al., 2018), hippocampal (Assari, 2020, Hanson et al., 2011, Hanson et al., 2015, Judd et al., 2020, Noble et al., 2012), and global gray matter volume (Hanson et al., 2013, Luby et al., 2013, McDermott et al., 2019), and surface area (Brito et al., 2018, Judd et al., 2020, McDermott et al., 2019, Noble et al., 2015, Rakesh and Whittle, 2021, Merz et al., 2021). A recent study assessing relations between a broad range of cognitive, behavioral, clinical, psychosocial, and socioeconomic measures and child brain structure, found strongest relations between SES factors and complex multimodal patterns involving cortical thickness, area, and volume (Alnæs et al., 2020). Of note, SES is a concept consisting of partly distinct sub-factors, and each factor has been reported to show partly unique associations with brain structure (Rakesh and Whittle, 2021).

The cerebral cortex, with its characteristic folding pattern, comprises the outermost part of the cerebrum (Destrieux et al., 2010). It is considered to be a principal neural structure for human cognitive abilities (Harris and Shepherd, 2015, Rakic, 2009). The cortex shows a lengthy developmental trajectory, with regions supporting lower level sensorimotor abilities generally preceding maturation of regions supporting higher-order functions (Norbom et al., 2021, Sydnor et al., 2021). Although there are numerous reports of associations between parental SES and cortical macrostructure, no MRI based study has previously assessed cortical microstructure.

As detected from MRI, a prominent feature of the developing brain is an increase in its brightness, and assessments of such signal intensity variation in the T1-weighted (T1w) and T2-weighted (T2w) image, could provide a microstructural correlate to the reported relation between parental SES and youth cortical macrostructure. Although debated (Hagiwara et al., 2018, Ritchie et al., 2018, Uddin et al., 2019), the T1w/T2w ratio has been suggested as a proxy measure of cortical myelination (Glasser and Van Essen, 2011, Van Essen and Glasser, 2014) while having the benefit, as compared to quantitative relaxometry, of being based on conventional MRI sequences (Nerland et al., 2021). Cortical myelination is a crucial feature of postnatal brain development, allowing for efficient signal transmission and structural support (Bartzokis, 2012, Baumann and Pham-Dinh, 2001, Liu et al., 2019, Waxman and Bennett, 1972). Several studies substantiate the utility of T1w/T2w ratio for mapping global and regional cortical maturational patterns (Baum et al., 2021, Grydeland et al., 2013, Grydeland et al., 2019, Norbom et al., 2020, Shafee et al., 2015), with ratios being positively associated with age across childhood and adolescence, as well as for assessing associations with cognitive performance in youth (Grydeland et al., 2013, Norbom et al., 2020, Vandewouw et al., 2019).

Although the T1w/T2w ratio shows promise for capturing distinct patterns of cortical development, no previous study has tested the measure in relation to parental SES. To this end, we first assessed whether there is a relation between parental SES and youth cortical microstructure as measured by vertex wise T1w/T2w ratio. We also performed comparisons to standard morphometry, assessing relations to vertex wise cortical thickness and surface area. Second, due to previous reports of SES-brain relations interacting with age, we assessed whether the relations between parental SES and T1w/T2w ratio depended on age. Finally, we tested if individual differences in cortical microstructure mediated associations between parental SES and specific cognitive abilities in childhood and adolescence. We hypothesized positive associations between parental SES and T1w/T2w ratio within frontal regions, as this has been reported for surface area (Noble et al., 2015). Next, based on previous cortical and subcortical assessments of the PING sample, we anticipated that the relations between parental SES and T1w/T2w ratio would interact with participant age, so that effects are stronger for younger participants (Merz et al., 2018, Piccolo et al., 2016). Finally, we hypothesized that the association between parental SES and cognitive abilities is partly mediated by T1w/T2w ratio, i.e., an average causal mediation effect.

## 2 Materials and methods

### 2.1 Participants

The current study investigated participants from the publicly available Pediatric Imaging, Neurocognition, and Genetics (PING) study (http://ping.chd.ucsd.edu), which consists of typically developing youths aged 3–21 years, with available behavioral and genetic data, as well as multimodal imaging for a large subgroup (Akshoomoff et al., 2014, Jernigan et al., 2016). Participants were requited through local initiatives from the metropolitan areas of Baltimore, Boston, Honolulu, Los Angeles, New Haven, New York, Sacramento, and San Diego in the USA. Exclusion criteria were major developmental, psychiatric (except ADHD) or neurological disorders, head trauma, pregnancy, prematurity, severe prenatal exposure to illicit drugs or alcohol, and contraindications for MRI. Written informed consent was attained for all participants 18 years of age or older, while written parental informed consent was additionally attained for younger participants.

**Fig. 1:**
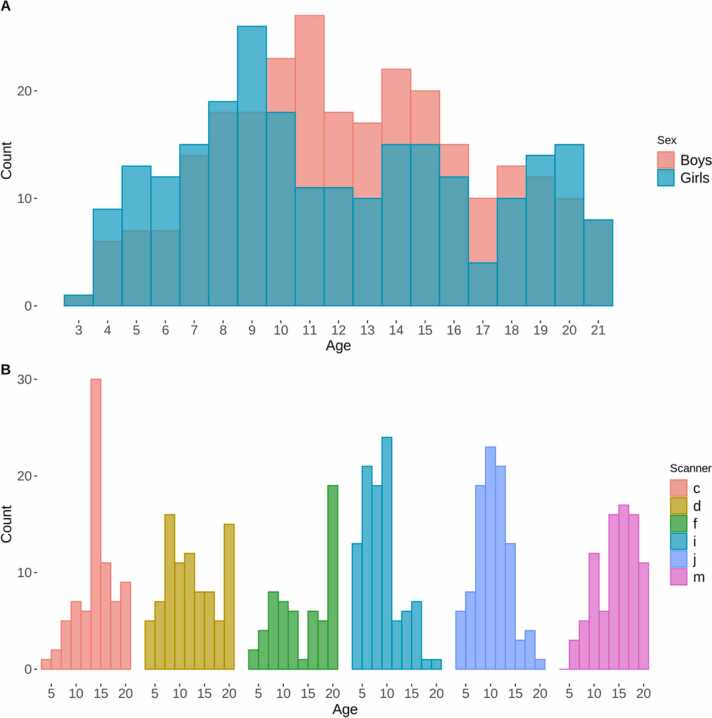
Age and sex distribution of sample (*n* = 504). Plot (A) depicts the age and sex distribution of the final sample, while plot (B) depicts the age distribution within each scanner.

From 998 participants with available T1w MRI data, 322 participants were excluded due to missing or insufficient image resolution (voxel size >1.2 mm) of T2w data. 55 participants were subsequently excluded during quality control (QC) as described below, another 90 participants were excluded due to missing socioeconomic information, 16 participants due to missing genetic ancestry information, and 11 participants from a single scanner were removed. This yielded a final sample of 504 participants (238 female) aged 3–21 years (mean = 12.2, SD = 4.7) See Fig. 1A and Supplementary Table 1 for further information on sample demographics. Four participants had completely missing cognitive test data and were excluded from relevant analyses.

### 2.2 Genetic ancestry factors (GAFs)

DNA extraction and genotyping from saliva samples were performed by Scripps Translational Research Institute (STRI), as described in detail elsewhere (Bakken et al., 2012). PING ancestry assessments were performed using a supervised clustering approach, and resulted in six continuous variables, each corresponding to major continental populations as defined by a reference panel created from publicly available data of individuals of known global ancestry (Alexander and Lange, 2011). The six GAFs were labeled African, Central Asian, East Asian, European, Native American, and Oceanic.

### 2.3 Measurements of socioeconomic status

Parental SES is challenging to quantify, and as no study have assessed relations to T1w/T2w ratio, we chose to calculate a single overarching composite score, using household income, guardian education and guardian occupation. This information was reported by a guardian or the participant themselves if aged 18 years or over, in the "PING Study Demographics and Child Health History Questionnaire” (Jernigan et al., 2016). As the guardian in most cases was either the biological (94.8 %), or adoptive/step- (2.8 %) parent of the child, we use the term “parental”.

**Fig. 2:**
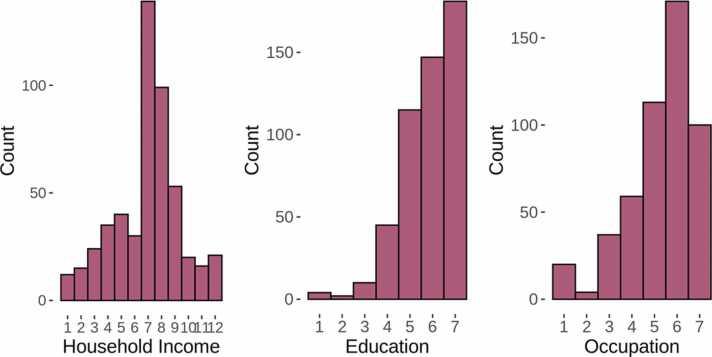
Distribution of raw SES measures in the current sample. The figure shows histograms of the distribution of household income, parental education, and parental occupation.

Total yearly family income was measured on a 12-point categorical scale (from <5000$ to>300.000$), while parental education and occupation was measured on separate 7-point scales (from <7 years of school to holding professional degree such as MA/MD/Ph.D., and unskilled employee to higher executive/major professional, respectively) (Khundrakpam et al., 2020). In order to include as many participants and as much data as possible, we used the highest reported education and occupation of either parent. The distribution of each specific SES measure in the current sample is shown in Fig. 2 and further details are reported in Supplementary Tables 2–4. Income and education, which were reported based on categorical ranges, were re-coded as the midpoint in dollars and approximate total years of schooling, respectively, then income was log10 transformed due to a positively skewed distribution (skewness = 1.32), and lastly, the SES variables were z-standardized.

**Fig. 3:**
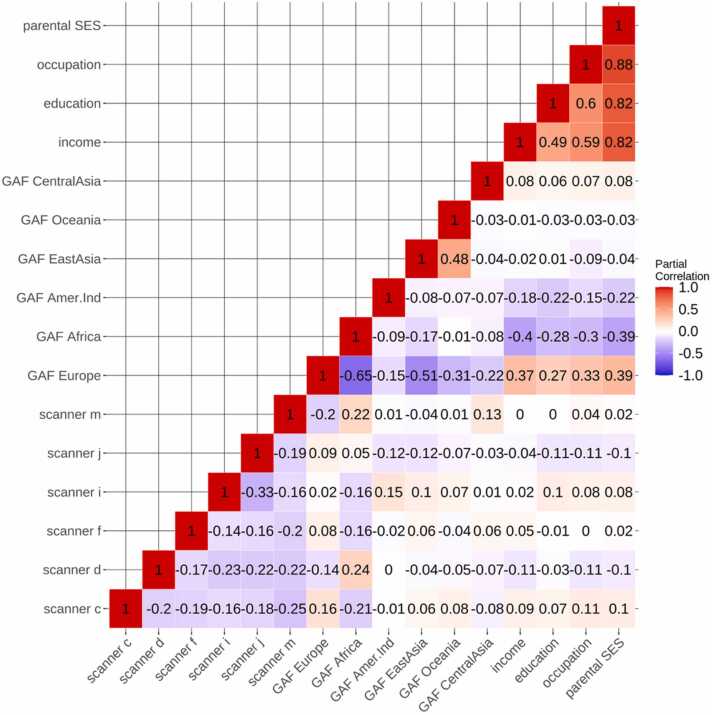
Partial correlation matrix of SES relations. The figure shows a partial correlation matrix of the relation between SES metrics, scanner, and GAFs, while controlling for age.

To calculate the composite score, using all subjects from our MRI sample with accessible SES data (n = 531); household income, parental education and parental occupation were included in a principal component analysis (PCA) using the prcomp package in R. The first component explained 70.9 % of the variance and loadings were z-normalized, inverted so that higher loading represented higher SES, and extracted as a general measure of parental SES. PCA details are shown in Supplementary Fig. 1, and a partial correlation matrix, controlling for age, between SES measures, scanner and GAFs is shown in Fig. 3.

### 2.4 MRI acquisition and processing

For the current study, imaging data were attained on six separate 3T scanners from two different manufacturers; GE medical systems (Signa HDx and Discovery MR750) and Siemens (TrioTim). Fig. 1B depicts scanner specific age distributions. Across sites, the scan protocol included a 3D T1w inversion prepared RF-spoiled gradient echo scan, and a 3D T2w variable flip angle fast spin echo scan (voxel sizes ≈ 0.9–1.2 mm^3^), both acquired with prospective motion correction (PROMO) (Jernigan et al., 2016, White et al., 2010). Care and safety procedures included exposure and habituation to the MRI machine and environment while accompanied by a parent or technician, additional head padding for the youngest individuals, and behavioral support including breaks in between scans if needed. Participants were presented with a movie of their choice during the structural protocol, and no participants were sedated for imaging (Brown and Jernigan, 2012).

Imaging data were processed using the three step HCP pipeline (Glasser et al., 2013). Briefly, for each subject, step 1 produces an undistorted native space, aligns the T1w and the T2w image, performs bias field correction, and register the native- to MNI space. Then, with certain default setting modifications of FreeSurfer (Fischl, 2012) version 6.0, step 2 performs volume segmentations, surface reconstructions of amongst others the white- and pial surface and registers all surfaces to the common template (fsaverage). Cortical thickness is computed as the shortest vertex-wise distance between the white and pial surface, while surface area is computed by the amount of vertex-wise expansion and contraction needed for the white surface to fit fsaverage (Dale et al., 1999, Fischl et al., 1999). Finally, step 3 produces NIFTI volume- and GIFTI surface files, including the T1w/T2w ratio maps as based on methods by Glasser and Van Essen (2011). First, the T1w volume is divided by T2w volume. Then, all resulting cortical ribbon voxels are selected by a cylinder with a height and radius of the local cortical thickness. Voxels suspected of being highly affected by blood vessel- and CSF partial voluming effects (T1w/T2w ratio > ± 1 *SD* of all ribbon values) are excluded, and, centering the cylinder on each vertex of a native surface called midthickness, the remaining voxels are mapped onto it and averaged using a Gaussian weighted function to produce a single value per vertex. The medial wall is assigned values of zero. The final outputs include standard, as well as bias-corrected, non-smoothed and smoothed (full width at half maximum = 4 mm) maps.

### 2.5 Quality control of imaging data

All T1w and T2w NIFTI images were QCed using MRIQC (Esteban et al., 2017). For each image, this pipeline returns both a binary flagging, and a dimensional quality index, based on measures of noise, spatial and tissue distribution, artifacts such as motion, and sharpness (Esteban et al., 2017). Then, a single trained rater visually inspected flagged datasets, and subjects were either included, tagged for detailed re-assessment post T1w/T2w ratio map creation, or excluded unless replacement with another satisfactory run was possible. The remaining images were subsequently processed through the HCP pipeline. Finally, all T1w/T2w ratio maps were visually inspected by the same trained rater, and subjects with poor maps were excluded. The 55 subjects excluded during this process were demographically similar to the larger included sample (Supplementary Table 1).

### 2.6 Assessment of cognitive abilities

Cognitive abilities were assessed using the computerized NIH Toolbox Cognition Battery (Akshoomoff et al., 2014, Weintraub et al., 2013, Weintraub et al., 2013). This assessment consists of seven tasks, measuring eight key cognitive abilities and is presumed to be valid within the age range of the current study (Weintraub et al., 2013, Weintraub et al., 2013). See Table 1 and Supplementary materials for an overview of the tests and in-detailed description of each test, respectively. We used the sum scores of each test, which were calculated and described in detail elsewhere (Akshoomoff et al., 2014) and performed z-standardization. Keeping as much data as possible, we imputed 72 missing cognitive scores from 58 subjects in a slightly larger sample (n = 531) in R, using the “mice” package (Buuren and Groothuis-Oudshoorn, 2011), which in our final sample translated to 41 imputed values for 17 subjects. Also, as previously described, four participants in our final sample had completely missing cognitive data, resulting in a sample size of 500 participants for all statistical tests involving cognitive scores.

**Table 1:** Cognitive tests. The table shows cognitive tests from the computerized NIH Toolbox Cognition Battery, in order of presentation to subjects, and the dimensions tested.

Test	Cognitive dimension
Dimensional change card sort test	Cognitive flexibility
Flanker inhibitory control and attention test	Inhibitory control Visual attention
Picture sequence memory test	Episodic memory
Pattern comparison processing speed test	Processing speed
Oral Reading recognition test	Oral reading skill
List sorting working memory test	Working memory
Picture vocabulary test	Vocabulary knowledge

### 2.7 Statistical analyses

Relationships between parental SES and vertex wise T1w/T2w ratio were tested using linear models as implemented in the Permutation Analysis of Linear Models (PALM) toolbox (Winkler et al., 2014). First, we tested for the main effect of SES on T1w/T2w ratio covarying for age, sex, continuous GAF scores, and scanners dummy coded as 6 separate variables. In order to compare possible relations with commonly studied measures of cortical morphometry, we also tested the main effect of SES on vertex-wise cortical thickness and vertex wise cortical surface area using the same model setup. Second, using PALM, we tested for interactions between SES and age on vertex-wise T1w/T2w ratio, adding sex, GAFs, scanners and the main effect of SES and age as covariates.

To robustly assess statistical significance without holding assumptions of data distribution, we shuffled the data using 10,000 permutations. Since each scanner should be considered as a separate variance group, block exchangeability restrictions were added so that data was shuffled within each scanner only. Thus, instead of t-statistics, the Aspin-Welch v-statistic is outputted. Moreover, to control for multiple comparisons, we employed family wise error (FWE) correction with threshold-free cluster enhancement (Smith and Nichols, 2009), and corrected across each contrast, and both hemispheres, using a significance threshold of *p* < .05.

Finally, we tested if T1w/T2w ratio mediated associations between parental SES and specific youth cognitive abilities. We first tested for possible relation between SES and eight specific cognitive abilities using z-standardized sum scores and linear models, with age, sex, and six GAFs included as covariates. The false discovery rate (FDR) was adjusted using the Benjamini-Hochberg procedure and using a significance threshold of *p* < .05. Then, cognitive scores showing a significant association with SES were included in separate mediation analyses using the package “mediation” in R. Here, SES was added as an independent variable, and cognitive ability as a dependent variable. The regions showing a significant relation in the initial parental SES-T1w/T2w ratio analyses were averaged within hemisphere and added as mediator in separate models. Age, sex, GAFs (and scanner when imaging data was assessed) were added as covariates, and nonparametric bootstrapping was employed using 1000 Monte Carlo draws. Mean T1w/T2w ratio from relevant regions were extracted using the “metric-stats” command from the HCP workbench. More specifically, individual T1w/T2w ratio maps were masked by the significance map outputted by PALM from the main effect SES-T1w/T2w analysis, and mean T1w/T2w ratio was extracted from these regions for each hemisphere separately.

Analyses of each SES indicator (income, education and occupation) on T1w/T2w ratio, total surface area, and mean cortical thickness are described and reported in the Supplementary materials.

## 3 Results

### 3.1 Relations between parental SES and cortical T1wT2w ratio

Permutation testing revealed significant negative associations between SES and T1w/T2w ratio, indicating that lower SES was associated with higher ratio, in widespread frontal-, insular-, superior temporal-, medial parietal- and occipital regions (Fig. 3). The associations showed a left hemispheric spatial dominance. Supplementary analyses of each SES sub-factor on T1w/T2w ratio revealed similar findings for parental education and occupation (Supplementary Figure 2–3), but no significant associations for family income, indicating that youths who have parents with lower education or occupation have higher T1w/T2w ratio, as compared to having parents with higher educational and occupational attainment. Concordant results were also found for mean T1w/T2w ratio (Supplementary Table 5, Supplementary Figure 4).

### 3.2 Relations between parental SES and cortical morphology

To compare the T1w/T2w ratio results with cortical morphometry, we next performed similar analyses assessing associations between parental SES and vertex-wise cortical thickness and surface area. The analyses revealed no significant associations between parental SES and cortical thickness or cortical surface area. Supplementary analyses were in concordance with vertex-wise results, i.e., no significant associations were found between parental SES or its indicators (family income, parental education or parental occupation) and mean cortical thickness or total surface area (Supplementary Table 5, Supplementary Fig. 4). Z-tests revealed significantly larger effect sizes for mean T1w/2w ratio as compared to mean cortical thickness (z-statistic = −2.42, corrected p = .046), but not compared with total surface area (z-statistic = −0.8, corrected p = .426) (Supplementary Table 6).

### 3.3 Interactions between parental SES and youth age on T1wT2w ratio

**Fig. 4:**
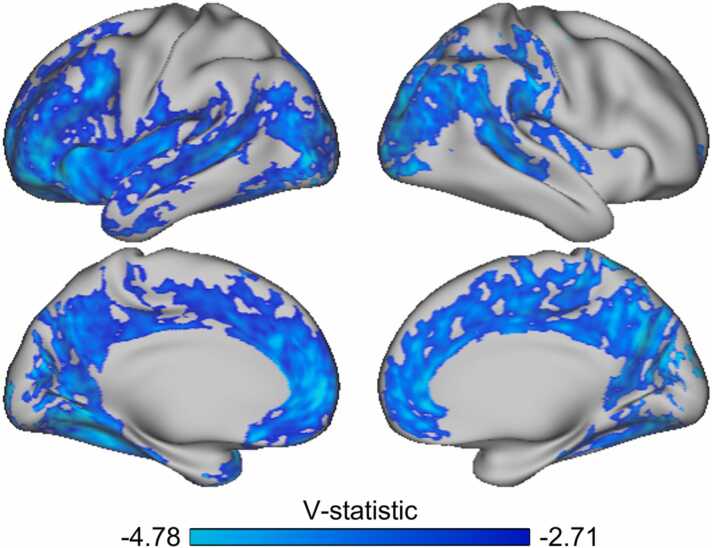
Relations between parental SES and youth T1w/T2w ratio. The figure shows a v-statistics map, masked by the familywise error corrected significance map with a threshold of > = 1.6 log-p (thus including corrections across hemispheres), of the association between parental SES and youth T1w/T2w ratio. Cold colors represent a negative association between parental SES and T1w/T2w ratio.

We next tested whether the relation between parental SES and vertex-wise T1w/T2w ratio was dependent on age. The results showed no significant interaction effect between parental SES and subject age on vertex wise T1w/T2w ratio Fig. 4.

### 3.4 T1w/T2w ratio as a potential mediator of the parental SES- cognitive ability relations

We finally tested possible relations between parental SES and eight cognitive abilities in youths and whether T1w/T2w ratio could mediate these relations. Parental SES was positively related to reading (*β* = 0.14, t = 4.94, corrected p < .001) and vocabulary (*β* = 0.17, t = 5.67, corrected p < .001), indicating that children and adolescents from lower SES families perform poorer on tasks measuring language abilities. We found no significant relations between parental SES and performance on the other cognitive tests (see Supplementary Table 7). Reading and vocabulary were then included as dependent variables in separate mediation analyses with parental SES and mean ROI based T1w/T2w ratio as independent and mediator variable, respectively. There was no significant mediation effect of T1w/T2w ratio on the SES-reading or SES-vocabulary relationship (see Supplementary Table 8).

## 4 Discussion

We assessed the relation between parental SES and cortical microstructure as reflected by T1w/T2w ratio in youth and found widespread negative associations that appeared stable across childhood and adolescence. These neurobiological differences did not however mediate SES related differences in reading and vocabulary knowledge. Moreover, we found no relations between parental SES and cortical thickness or surface area, suggesting that T1w/T2w ratio might be a particularly sensitive measure for assessing SES-brain relations.

Parental SES was negatively associated with youth T1w/T2w ratio, indicating that children and adolescents of families with lower SES have higher ratios, particularly in frontal, temporal and occipital regions, that is most widespread in the left hemisphere. As hypothesized, some of the strongest relations between parental SES and T1w/T2w ratio were within frontal cortical regions. The intracortical myelination of the frontal lobe shows an extended developmental trajectory (Nieuwenhuys, 2013, Yakovlev and Lecours, 1967), and it is plausible that cortical regions with wider developmental windows are particularly sensitive for accumulating environmental influences. We also found associations within the temporal lobe, which also shows a particularly lengthy myelination process (Bartzokis, 2004). On the other hand, we also found relations with T1w/T2w ratio within the visual cortex, a region known to extensively myelinate during the first years of life (Miller et al., 2012, Yakovlev and Lecours, 1967). This suggests associations between parental SES and microstructural properties of both early and late developing cortical regions. The spatial distribution of our findings partly overlap with previous findings reported between SES and cortical surface area (Rakesh and Whittle, 2021).

The negative association between parental SES and T1w/T2w ratio is counterintuitive. Our results indicated that children and adolescents in lower SES families have a higher T1w/T2w ratio. As it has been shown that T1w/T2w ratio appear to globally and steadily increase from 3 years of age and until young adulthood (Grydeland et al., 2019, Norbom et al., 2021), one could infer that youths from lower SES families appear to have a more developed cortex. Although this is the first assessment of T1w/T2w ratio in relation to SES, previous studies have found negative associations between T1w/T2w ratio and cognition in youth. For instance, Grydeland and colleagues (2013) reported lower ratios with greater cognitive performance stability in youths aged 8–19 years, and a prior study using the PING sample found negative associations between T1w/T2w ratio and general and several specific cognitive abilities (Norbom et al., 2020). Possible reasons for the direction of findings are fourfold. First, excess levels of cortical myelin beyond a normative range could be disadvantageous due to its inhibitory qualities for neural plasticity (Snaidero and Simons, 2017). Second, separate tissue properties besides myelin may account for outcome variations related T1w/T2w ratio, particularly in regions known to have low levels of myelin in youth (Norbom et al., 2020). Third, misclassification of deep cortical matter by imaging analysis software’s, thereby resulting in an underestimation of the true cortical thickness, could yield lower ratio in association with beneficial outcomes (Norbom et al., 2020). Finally, corroborating a recognized theory within social science, exposure to poverty and other early life adversaries, can foster accelerated maturation, as compared to their lower exposed peers (Belsky, 2019, Colich et al., 2020). Such patterns have been found previously using multiple biological- and MRI based metrics (Colich et al., 2020). Future studies should use quantitative relaxometry to further probe the underlying neurobiology of SES related links to T1w/T2w ratio, ideally combined with a prospective longitudinal design for assessing the impact of early life exposures on individual brain microstructural development over time. Indeed, a recent study assessed the association between neighborhood poverty, parental education and occupation and magnetization transfer (MT) in youths aged 14–25 year. They found that living in a more deprived neighborhood before age 12 was robustly linked with lower MT in adolescence, interpreted as slower myelin growth, in multiple-, including sensorimotor cortical regions. Cortical findings were somewhat reduced when adding parental occupation as a co-variate, while current economic disadvantage and parental occupation was not associated with MT (Ziegler et al., 2020).

There was no evidence for associations between parental SES and T1w/T2w ratio interacting with age. Our findings are thus not in line with previous research reporting that SES has a stronger impact on younger-, as compared to older brains (Merz et al., 2018, Piccolo et al., 2016). Still, due to our sample consisting of few children within the youngest age brackets, the statistical power to detect such an interaction is limited and caution is thus warranted when interpreting these results.

Parental SES and T1w/T2w ratio was associated within regions known to be highly relevant for higher-order cognitive functions. Moreover, the left hemisphere spatial dominance could signify links to language abilities, which have been extensively documented as varying with parental SES (Schwab and Lew-Williams, 2016). For instance, the increase in vocabulary is slower for children from lower-SES families than other children (Arriaga et al., 1998, Morgan et al., 2015), presumably due to both quantitative and qualitative exposure differences, with reports of young affluent children by 4 years of age hearing on average about 30 million more words than children from less affluent families (Hart and Risley, 1995), with higher lexical diversity and conversational fluency (Huttenlocher et al., 2010, Rowe, 2008, Rowe, 2012). Congruently, we found that children and adolescents of parents with lower SES were poorer readers and had poorer vocabulary knowledge. Such SES-related variability in language abilities must somehow transit the brain. Nevertheless, counter to our hypothesis, we found no indication of microstructural differences explaining socioeconomic differences in language abilities. This could either indicate that our microstructural findings are related to other untested outcomes, or that we had insufficient power to detect such mediatory effects. Indeed, mediation was only assessed in regions shown to be significant in our main SES-T1w/T2w ratio analysis, and ratio values were, within each hemisphere, averaged across these regions, yielding subpar specificity. As we demonstrate parental SES- T1w/T2w ratio associations, future studies should attain higher statistical power to perform more elaborate and vertex-wise mediation assessments, further tapping into what relations could be relayed.

The T1w/T2w ratio has been presumed to indirectly reflect differences in intracortical myelin content (Glasser and Van Essen, 2011, Salat et al., 2009), based on the notion that macromolecules in the myelin sheath, including cholesterol, has a major influence on T1 longitudinal- and T2 and T2 * transverse relaxation times (Does, 2018, Koenig, 1991, Koenig et al., 1990). Other signal influences, include iron (Miot-Noirault et al., 1997, Stüber et al., 2014) and water concentration (Miot-Noirault et al., 1997). Although several human histological and quantitative relaxometry studies support T1w/T2w ratio as being a decent myelin proxy (Eickhoff et al., 2005, Nakamura et al., 2017, Shafee et al., 2015), other recent attempts have not revealed consistent links between T1w/T2w ratio and either myelin-related genes or other standard MRI-based myelin proxies. This includes moderate correlations between T1w/T2w ratio and MT in gray matter (Hagiwara et al., 2018), and low correlations with myelin water fraction both in gray and white matter (Uddin et al., 2019). A combined post-mortem and neuroimaging study reported that other molecular properties were more strongly associated with T1w/T2w ratio than myelin associated genes (Ritchie et al., 2018). Moreover, T1w/T2w ratio has been found to show a strong correlation with dendrite density (Righart et al., 2017). Together, these findings demonstrate that T1w/T2w ratio is by no means a straightforward myelin proxy.

There was no relation between parental SES and youth cortical morphometry as assessed by cortical thickness and surface area. In line with a recent review (Rakesh and Whittle, 2021), vertex wise analysis revealed no relation between parental SES and cortical thickness. Counter to previous findings (Brito et al., 2018, Judd et al., 2020, McDermott et al., 2019) and our hypothesis, however, we also did not find an association between parental SES and surface area. A previous study using an overlapping and larger sample than the current study, reported a logarithmic association between family income-, and a linear association with parental education and global cortical surface area in youths, which partly mediated socioeconomic differences in certain cognitive abilities (Noble et al., 2015). Our supplementary analyses on each sub-factor of SES and total surface area did not support these findings, and instead we show that intensity metrics perhaps are more sensitive than cortical morphology when examining the association of parental SES on youth brain development. Indeed, statistical tests revealed stronger effects for mean T1w/T2w ratio as compared to mean cortical thickness, albeit no difference between mean T1w/T2w ratio and total surface area, or mean cortical thickness and total surface area. There are multiple discrepancies between the current study and Noble et al. (2015). First, we had a smaller sample (n = 504 vs. n = 1099), subsequently limiting our power to detect the previously reported effects. A power analysis of the current sample size revealed a power of 0.84 for finding β = 0.14 if present, while Noble et al. had a power of 0.99. Beyond the arbitrary significance threshold, there is inherent uncertainty of estimates within both studies as sample sizes were relatively small, also, effects might change depending on the subset of PING used. Second, we assessed parental educational attainment as highest attained- and not average attained education as used in Noble et al. Third, for MRI quality control, we used the data driven software MRIQC on raw-, and visual inspection of raw and processed data excluding 55 subjects, while Noble et al. performed a “standard quality-image check” without further details except all images passing quality assessment. Fourth, we used the HCP-pipeline based on Freesurfer 6.0, while Noble et al. used a modified Freesurfer suite. Fifth, the current study used linear models in PALM for vertex-wise analyses and R for total metrics, while Noble et al. employed general additive modeling, with details regarding knots and splines unspecified. We believe the sum of these differences, contribute to the divergent findings.

SES is a construct consisting of partly distinct sub-factors. Concordantly, each aspect of SES have been reported to show somewhat unique associations with brain structure (Rakesh and Whittle, 2021). Our reasoning for choosing a composite score for quantifying SES was twofold. First, composite scores generally show stronger relations with brain metrics (Rakesh and Whittle, 2021), and second, a composite score mimics the actual SES construct better than any subcomponent alone. Beyond our overarching parental SES findings, specific assessments of each sub-component revealed similar relations between cortical microstructure and parental occupation, and highly spatially widespread associations with parental education. This indicates that the development of cortical microstructure in childhood and adolescence appear to be more closely linked to parental position within the social hierarchy (Khundrakpam et al., 2020), and parent to child stimulation such as cognitive-, and language related input respectively (Duncan and Magnuson, 2012). In our study, family income, a suggested proxy for access to material resources (Duncan and Magnuson, 2012), was not significantly associated with youth cortical microstructure. Future studies should further examine which specific SES factors that are particularly relevant for child brain development, as well as the mechanisms underlying these effects, information which could be valuable from a sociopolitical awareness and targeting standpoint.

SES is a broad multidimensional concept. One could argue that our component was too narrow, disregarding factors like neighborhood metrics which capture variability related to crime, pollution, greenery, and access to libraries and so on (Farah, 2017). There are also subjective and cultural aspects to SES, and in this context the genetic origin of the participants may be less central as compared to their self-reported and subjective ethnic identification and how they are perceived by others. Importantly, a number of twin and family studies also show that SES is highly heritable (Ørstavik et al., 2014, Tambs et al., 2012) and recent GWAS studies indicate that heritable phenotypes such as intelligence might link molecular genetic inheritance and phenotypic gradients in SES (Hill et al., 2019). Therefore, future studies could use genetically informed designs and intervention studies to test the relative importance of social causation versus selection processes on child brain structure and function.

It should be noted that we in the current study were not able to perform radiofrequency transmit field (B1 +) correction, as proposed in a recent preprint (Glasser et al., 2022) and that such biases could be correlated with our variables of interest.

To conclude, we report widespread and age-stable associations between parental SES and cortical microstructure in childhood and adolescence. We also found relations between parental SES and youth language abilities, but cortical microstructure did not mediate these associations. Probing the neurobiology underlying the associations between parental SES, its partly distinct sub-factors and central life outcomes in childhood in adolescence may provide new ways to understand and eventually reduce societal disparities. The results of the present study suggest that T1w/T2w ratio might be a particularly sensitive neuroimaging metric for capturing SES-related differences in the child brain.

## Data_statement

The Pediatric Imaging, Neurocognition and Genetics Study (PING) provided data. The National Institutes of Health (Grant RC2DA029475), and the National Institute on Drug Abuse and the Eunice Kennedy Shriver National Institute of Child Health & Human Development funded collection and sharing. PING data are disseminated by the PING Coordinating Center at the Center for Human Development, University of California, San Diego. The data is open access upon a successful application to the PING study administrators.

## Declaration of Competing Interest

The authors report no conflicts of interest. OAA is a consultant to HealthLytix.

## Data Availability

Data will be made available on request.
