# Erratum to “Parental socioeconomic status is linked to cortical microstructure and language abilities in children and adolescents” [Dev. Cogn. Neurosci. 56C (2022) 101132]

**DOI:** 10.1016/j.dcn.2023.101225

**Published:** 2023-03-02

**Authors:** Linn B. Norbom, Jamie Hanson, Dennis van der Meer, Lia Ferschmann, Espen Røysamb, Tilmann von Soest, Ole A. Andreassen, Ingrid Agartz, Lars T. Westlye, Christian K. Tamnes

**Affiliations:** a PROMENTA Research Center, Department of Psychology, University of Oslo, Norway; b NORMENT, Institute of Clinical Medicine, University of Oslo, Norway; c Department of Psychiatric Research, Diakonhjemmet Hospital, Oslo, Norway; d Learning Research and Development Center University of Pittsburgh, USA; e Department of Psychology, University of Pittsburgh, USA; f School of Mental Health and Neuroscience, Faculty of Health, Medicine and Life Sciences, Maastricht University, the Netherlands; g K.G Jebsen Center for Neurodevelopmental Disorders, University of Oslo, Norway; h NORMENT, Division of Mental Health and Addiction, Oslo University Hospital & Institute of Clinical Medicine, University of Oslo, Norway; i Department of Psychology, University of Oslo, Norway; j Norwegian Institute of Public Health, Norway; k Centre for Psychiatry Research, Department of Clinical Neuroscience, Karolinska Institutet & Stockholm Health Care Services, Stockholm, Stockholm Region, Sweden

The publisher regrets that Dr Røysamb should have been the only Author affiliated with Norwegian Institute of Public Health, Norway.

The correct affiliations are given below.

Linn B. Norbom ^a b c^, Jamie Hanson ^d e^, Dennis van der Meer ^b f^, Lia Ferschmann ^a^, Espen Røysamb ^a i j^, Tilmann von Soest ^a^, Ole A. Andreassen ^g h^, Ingrid Agartz ^b c g k^, Lars T. Westlye ^g h i^, Christian K. Tamnes ^a b c^.

^a^ PROMENTA Research Center, Department of Psychology, University of Oslo, Norway.

^b^ NORMENT, Institute of Clinical Medicine, University of Oslo, Norway.

^c^ Department of Psychiatric Research, Diakonhjemmet Hospital, Oslo, Norway.

^d^ Learning Research and Development Center University of Pittsburgh, USA.

^e^ Department of Psychology, University of Pittsburgh, USA.

^f^ School of Mental Health and Neuroscience, Faculty of Health, Medicine and Life Sciences, Maastricht University, The Netherlands.

^g^ K.G Jebsen Center for Neurodevelopmental Disorders, University of Oslo, Norway.

^h^ NORMENT, Division of Mental Health and Addiction, Oslo University Hospital & Institute of Clinical Medicine, University of Oslo, Norway.

^i^ Department of Psychology, University of Oslo, Norway.

^j^ Norwegian Institute of Public Health, Norway.

^k^ Centre for Psychiatry Research, Department of Clinical Neuroscience, Karolinska Institutet & Stockholm Health Care Services, Stockholm Region, Stockholm, Sweden.

The publisher would like to apologise for any inconvenience caused.

