# Supplementary material for: Parental socioeconomic status is linked to cortical microstructure and language abilities in children and adolescents
Source: Dev Cogn Neurosci. 2022 Jul 4;56:101132. doi: 10.1016/j.dcn.2022.101132 (PMC9284438; doi:10.1016/j.dcn.2022.101132)
Supplement: Supplementary file 1 — Supplementary material. [file mmc1.docx]

**Supplementary Material:**

**Parental socioeconomic status is linked to cortical microstructure and language abilities in children and adolescents**

Linn B. Norbom, PhD, Jamie Hanson, PhD, Dennis van der Meer, PhD, Lia Ferschmann, PhD, Espen Røysamb, PhD, Tilmann von Soest, PhD, Ole A. Andreassen, MD, PhD, Ingrid Agartz, PhD, Lars T. Westlye, PhD, Christian K. Tamnes, PhD

**1 Introduction**

In our first set of supplementary analyses, we in separate models assessed the associations between family income, parental education, parental occupation, and youth cortical microstructure as measured by vertex wise T1w/T2w ratio. We expect positive associations within frontal regions, possibly extending into temporal regions for parental education and household income (Noble et al., 2015), and generally with smaller effects sizes than for the composite parental SES score (Rakesh & Whittle, 2021).

In our second set of supplementary analyses, we assessed the association between parental SES and each sub-component, and total surface area, mean cortical thickness and mean T1w/T2w ratio. We expect positive associations between family income and total surface area, and between parental education and total surface area. Further, we expect no relations with mean cortical thickness (Noble et al., 2015), and although exploratory we hypothesize that there will be a positive relation between parental SES and mean T1w/T2w ratio.

**2 Materials and Methods**

|  | Included | Excluded | t/x^2^ | p |
| --- | --- | --- | --- | --- |
| Total n | 504 | 55 | - | - |
| Age in years | 3.2–21.0 (mean = 12.2, *SD* = 4.7) | 3.4–20.6 (mean = 10.8, *SD* = 5.4) | 1.9 | 0.062 |
| Sex | Female = 238  Male = 266 | Female = 24  Male = 29 | 0.02  0.02 | 0.901  0.901 |
| Genetic ancestry | Europe = 285 (56.4%)  Africa = 41 (8.1%)  Native American = 1 (0.2%)  East Asian = 26 (5.2%)  Oceanic = 0 (0%)  Central Asia = 6 (1.2%)  Mixed ancestry =145 (28.8%) | Europe = 29 (55.8%)  Africa = 5 (9.6%)  Native American = 0 (0%)  East Asian = 2 (3.8%)  Oceanic = 0 (0%)  Central Asia = 2 (3.8%)  Mixed Ancestry = 14 (26.9%) | 0.00  0.01  0.00  0.01  -  0.85  0.01 | 1  0.917  1  0.937  -  0.358  0.905 |
| Raw SES measures | Income = 1-12 (mean =7.0, *SD* =2.4)  Education = 1-7 (mean = 5.8, *SD* = 1.2)  Occupation = 1-7 (mean =5.3, *SD* =1.5) | Income = 1-12 (mean = 6.8, *SD* = 2.0)  Education = 1-7 (mean = 5.7, *SD* = 1.3)  Occupation = 1-7 (mean = 5.2, *SD* = 1.5) | 0.49  0.74  0.57 | 0.629  0.463  0.569 |

Supplementary Table 1. Sample demographics of quality control (QC)-excluded subjects. The table depicts subjects included and excluded during the MRI QC procedure. Within the table, participants with a genomic similarity >= 0.8 are classified as belonging to that ancestral population, while the remaining subjects are classified as mixed ancestry. Note that these groupings are for the table only, and that for all statistical analyses genetic ancestry is assessed using continuous variables. All numbers are based on subjects with complete data for the relevant variable thus for the excluded subjects age and sex are based on n=53, genetic ancestry scores n=52, and raw SES measures n= 49. For continuous data t-values are depicted (t), while for categorical data, chi square (x^2^) with Yates continuity correction is depicted, both statistics are accompanied by p values.

*2.1 Measurements of socioeconomic status*

See Supplementary Tables 2, 3 and 4 for scale levels of family income, parental education and parental occupation (Khundrakpam et al., 2020). See Supplementary Figure 1 for PCA related explained variance and contributions of each specific SES sub-factor. The Hollingshead scale is a prominent measurement of social status, and although there were no such metric or adequate data to fully re-create it within the PING dataset, our “Pseudo-Hollingshead” ((Highest education *3) + (Highest occupation *5)) showed a Pearson’s correlation of 0.94 with our PCA created SES score.

| Scale level | Description |
| --- | --- |
| 1 | <$5,000 |
| 2 | $5,000 - 9,999 |
| 3 | $10,000 - 19,999 |
| 4 | $20,000 - 29,999 |
| 5 | $30,000 - 39,999 |
| 6 | $40,000 - 49,999 |
| 7 | $50,000 - 99,999 |
| 8 | $100,000 - 149,999 |
| 9 | $150,000 - 199,999 |
| 10 | $200,000 - 249,999 |
| 11 | $250,000 - 299,999 |
| 12 | $300,000+ |

Supplementary Table 2. Measurement of family income. The table shows the scale levels for family income.

| Scale level | Description |
| --- | --- |
| 1 | Less than 7 years of school |
| 2 | 7–9 years of school |
| 3 | 10–11 years of school |
| 4 | High school graduate |
| 5 | 1–3 years of college (also business school) |
| 6 | 4‐year college graduate (BA, BS, BM) |
| 7 | Professional (MA, MS, MD, PhD, , and the like) |

Supplementary Table 3. Measurement of parental education. The table shows the scale levels for parental education.

| Scale level | Description |
| --- | --- |
| 1 | Unskilled employees |
| 2 | Machine operators and semi‐skilled employees |
| 3 | Skilled manual employees |
| 4 | Clerical and sales workers, technicians, and owners of little businesses (<2 employees) |
| 5 | Administrative personnel, owners of small businesses, and minor professionals |
| 6 | Business managers, proprietors of medium‐sized businesses, and lesser professionals |
| 7 | Higher executives of large concerns, proprietors, and major professionals |

Supplementary Table 4. Measurement of parental occupation. The table shows the scale levels for parental occupation.


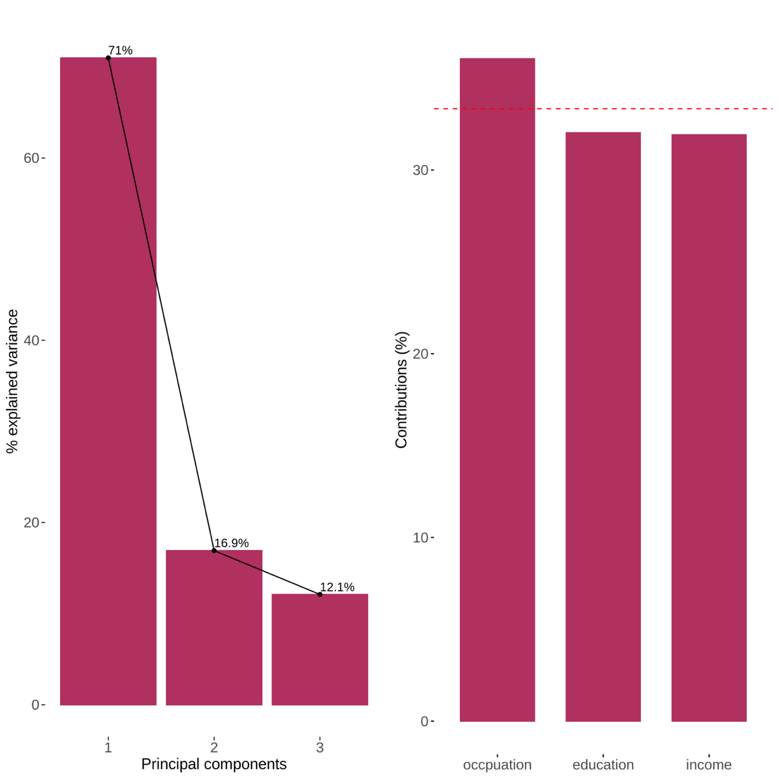


Supplementary Figure 1. Principal component analysis bar plots. The bar plot on the left-hand side shows the explained variance for each of the principal components, and the bar plot on the right-hand side shows the contribution of each specific SES sub-factor to first principal component chosen as the overarching SES measure.

*2.2 MRI acquisition*

Standardized multiple modality high-resolution protocols identical or close to identical to the pulse sequence parameters implemented at UC San Diego were installed across scanners. At UCSD, data were obtained on a GE 3T Signa HDx scanner and a 3T Discovery 750x scanner (GE Healthcare, Waukesha, WI) using eight-channel phased array head coils. The protocol included a sagittal 3D inversion recovery spoiled gradient echo (IR-SPGR) T1-weighted volume which was optimized for maximum gray/white matter contrast (TE = 3.5 ms, TR = 8.1 ms, TI = 640 ms, flip angle = 8°, receiver bandwidth = ± 31.25 kHz, FOV = 24 cm, freq = 256, phase = 192, slice thickness = 1.2 mm). It additionally included a sagittal 3D cube T2-weighted volume (TE = 69.3 ms, TR = 1500 ms, echo train = 40, FOV = 24 cm, freq = 256, phase = 192, slice thickness = 1.2 mm). Scanning durations were 8:05 and 4:25 minutes for the T1w and T2w sequence respectively (Brown & Jernigan, 2012). For further details we refer to previous publications (Brown & Jernigan, 2012; Jernigan et al., 2016; White et al., 2010).

*2.3 MRI quality assessment*

From 998 subjects with available MRI data, several were first directly excluded due to lack of a T2 sequence, for having unsatisfactory T1w or T2w resolution (> 1.2mm voxel size in any direction), and for having runs within the same folder, highly suspected of being of different individuals (n=322 in total). Subject folders could include several duplicate-, and unique NIFTI images from the same, and from different sessions, i.e. after re-positioning in the scanner, and all T1w and T2w NIFTIs were processed through the quality assessment pipeline MRIQC (Esteban et al., 2017). After visual inspection of flagged images (n=99) by a single experienced rater, flagged subjects were either included, tagged for re-assessment after T1w/T2w ratio map creation, or excluded due to poor image quality (n=17) when there was no option to replace the image with another satisfactory run. For non-flagged subjects with several images, the highest quality sequence was selected, based on having a superior “quality index” number outputted from MRIQC. The remaining subjects were consequently processed through the full Human Connectome Project (HCP) pipeline (Glasser et al., 2013), of which a few (n= 4) subjects failed completion and were excluded. Quality assessment of the T1w/T2w ratio maps were performed by careful visual inspection of previously tagged images by the same trained researcher, followed by inspection of lateral and medial snapshots of all T1w/T2w ratio maps. Several subjects were excluded at this final step due to poor T1w/T2w ratio maps (n=34), resulting in 621 subjects with complete MRI data.

*2.4 Cognitive assessment*

For in detailed descriptions of cognitive assessments see (Jernigan et al., 2016). In short, subjects had to match a picture to shape- or color-based targets during the “Dimensional Change Card Sort Test”, and the sum score was based on accuracy and reaction time incorporated by a two-vector method.

During the “Flanker Inhibitory Control and Attention Test”, presumed to measure both inhibition and attention, subjects had to assess symbol orientation while ignoring proximate and at times incongruent stimuli. While the youngest children were presented only with fish, subjects aged nine and above were presented smaller arrows. The attention score was based on congruent trials only, while the inhibition sum score was based on congruent and incongruent trials, both derived using a two-vector model.

The “Picture Sequence Memory Test” involved remembering sequences of pictured objects and activities in the presented order. The length of the sequences was age-depended, and the sum score was based on the total number of pairs (two following pictures) ordered correctly.

During the “Pattern Comparison Processing Speed Test” subjects were presented with pairs of pictures, and the sum score was based on total correct assessments of whether the pictures were identical or not. While subjects aged 8 and above pressed “yes” or “no” buttons, younger subjects correspondingly pressed a smiley or frowny face.

During the “Oral Reading Recognition Test” a word/letter was presented to read aloud. For pre-and low literacy readers, letters and other multiple-choice items were presented. The sum score was based on the total number of correct aloud readings.

The “List Sorting Working Memory Test” was a two-condition assignment where subjects had to remember a series of presented objects and repeat them in size order. The sum score was based on total correctly remembered objects repeated in size order across conditions.

Finally, the “Picture Vocabulary Test” consisted of responding to which picture most closely represented a word and the sum score was an item response theory theta conversion.

*2.5 Statistical analyses*

Associations between family income, parental education, parental occupation, and vertex wise T1w/T2w ratio were tested in separate linear models as implemented in the Permutation Analysis of Linear Models (PALM) toolbox (Winkler et al., 2014). For each model, age, sex, continuous GAF scores, and scanners dummy coded as 6 separate variables, were added as co-variates.

Attempting to corroborate previous findings where the PING sample was used (Noble et al., 2015), we next, in separate linear models in R, tested the associations between parental SES and its sub-factors, and total surface area, mean cortical thickness, and additionally mean T1w/T2w ratio. Age, sex, continuous GAF scores, and scanners were added as covariates. For surface area we ran additional analyses adding estimated total intracranial volume (eTIV) as an additional co-variate. The false discovery rate (FDR) was adjusted using the Benjamini-Hochberg Procedure and a significance threshold of *p* < .05. In order to test whether effect sizes outputted from the parental SES analyses only, were significantly different from each other, we computed the z-statistic (Clogg et al., 1995) for each pair of comparisons, resulting in 3 tests. The FDR was adjusted using the Benjamini-Hochberg Procedure and a significance threshold of *p* < .05

**3 Results**

*3.1 Relations between household income, parental education, parental occupation and T1w/T2w ratio*

Permutations revealed an almost global negative association between parental education and T1w/T2w ratio (Supplementary Figure 2), indicating that youths of parents with low education have higher ratio across most of the cortex, as compared to having parents with higher educational attainment. Moreover, maximum v-statistics were higher than for analyses employing the parental SES score.

Parental occupation showed a negative relation with T1w/T2w ratio, with a similar spatial distribution as findings from analyses using the parental SES score (Supplementary Figure 3). This indicates that children and adolescents of parents with lower occupation show higher ratio mainly in left frontal and right temporal-occipital regions as compared to having parents with higher occupational status.

We found no statistically significant effects of household income on vertex-wise T1w/T2w ratio.


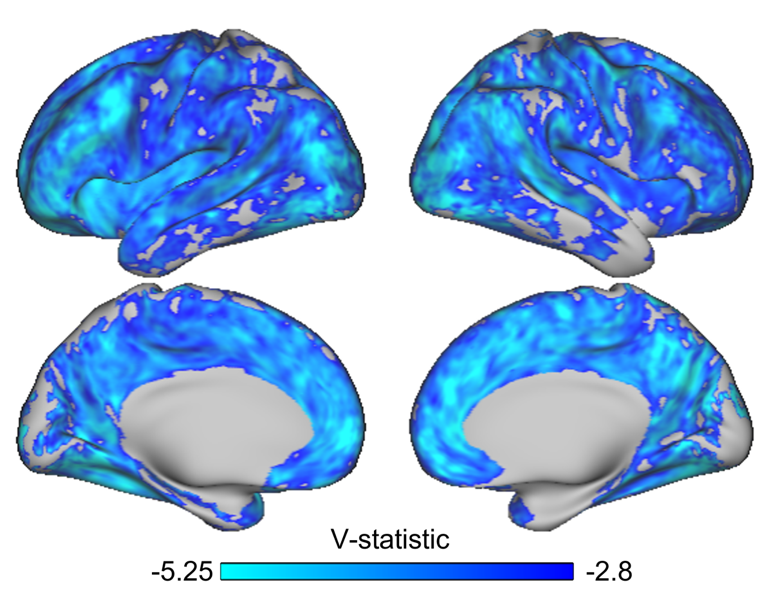


Supplementary Figure 2. Relations between parental education and youth T1w/T2w ratio. The figure shows a v-statistics map, masked by the familywise error corrected significance map with a threshold of >= 1.6 log-p (thus including corrections across hemispheres), of the relation between parental education and youth T1w/T2w ratio. Cold colors represent a negative association between parental education and T1w/T2w ratio.


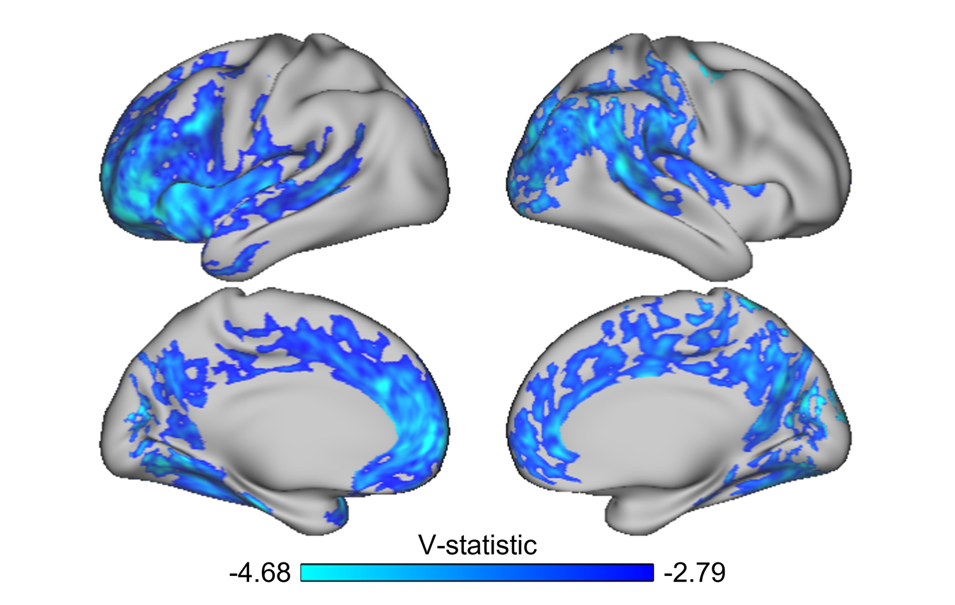


Supplementary Figure 3. Relations between parental occupation and youth T1w/T2w ratio. The figure shows a v-statistics map, masked by the familywise error corrected significance map with a threshold of >= 1.6 log-p (thus including corrections across hemispheres), of the relation between parental occupation and youth T1w/T2w ratio. Cold colors represent a negative association between parental occupation and T1w/T2w ratio.

*3.2 Relations between SES and total cortical area, mean cortical thickness and mean T1w/T2w ratio*

Converging with our vertex-wise findings, there were no significant relation between parental SES, nor sub-factors and total cortical surface area or mean cortical thickness. There were, however, associations between parental SES, parental education, parental occupation and mean T1w/T2w ratio (Supplementary Figure 4 and Supplementary Table 5). Re-running our total surface area analyses with eTIV as an additional co-variate resulted in a significant association between parental occupation and total surface area (Supplementary Table 6).


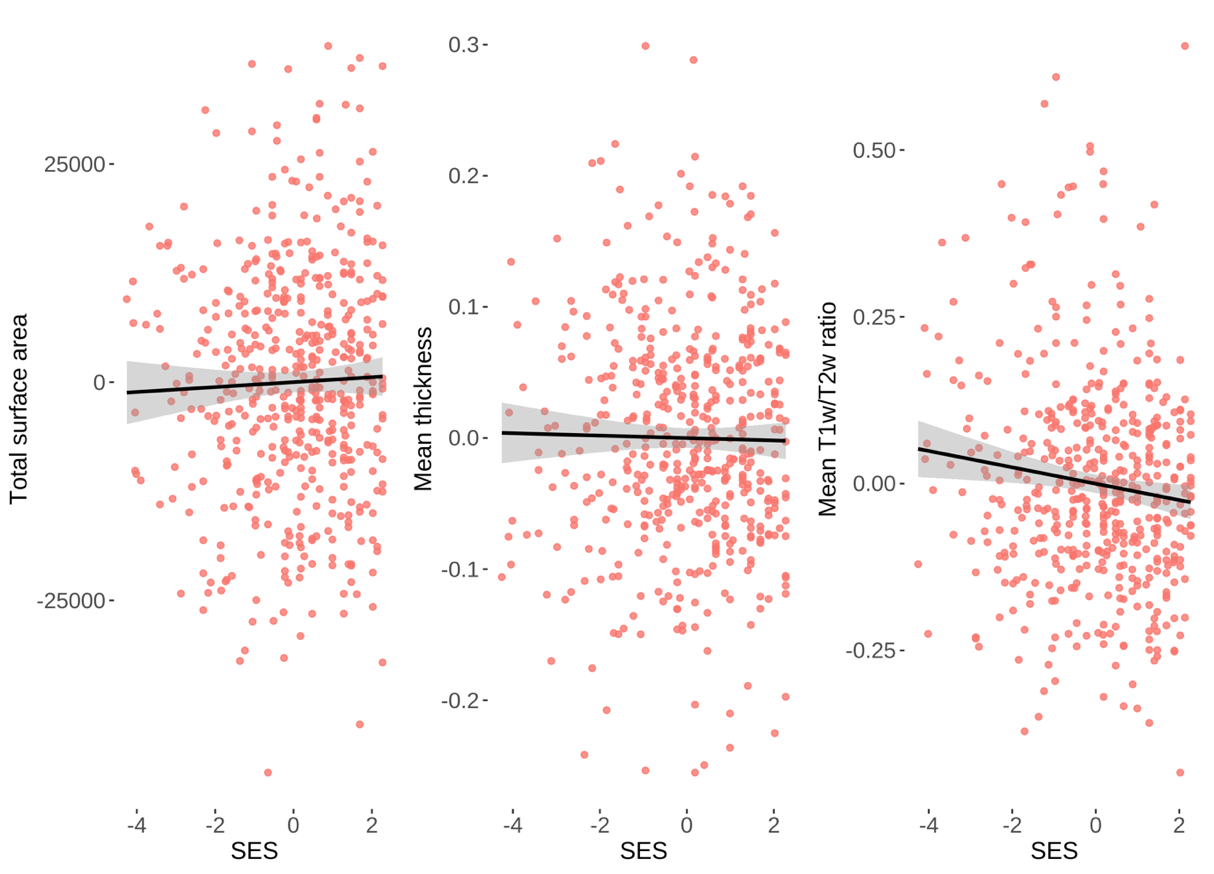


Supplementary Figure 4. Scatterplot of socioeconomic status (SES) and total cortical surface area, mean cortical thickness and mean cortical T1w/T2w-ratio. The figure shows SES plotted against total surface area, mean cortical thickness and mean T1w/T2w ratio. All brain metrics are residualized by age, sex, scanners and genetic ancestry factors (GAFs).

| Relationship | *β* | t | p | Corrected p |
| --- | --- | --- | --- | --- |
| Parental SES - total surface area | 0.03 | 0.80 | 0.426 | 0.933 |
| Education - total surface area | -0.01 | -0.16 | 0.872 | 0.933 |
| Occupation - total surface area | 0.02 | 0.49 | 0.623 | 0.933 |
| Income - total surface area | 0.07 | 1.63 | 0.104 | 0.933 |
| Parental SES - mean cortical thickness | -0.01 | -0.41 | 0.684 | 0.933 |
| Education - mean cortical thickness | 0.01 | 0.28 | 0.783 | 0.933 |
| Occupation - mean cortical thickness | -0.04 | -1.12 | 0.262 | 0.933 |
| Income - mean cortical thickness | 0.00 | -0.08 | 0.933 | 0.933 |
| Parental SES - mean T1w/T2w ratio | -0.05 | -2.98 | 0.003* | 0.033* |
| Education - mean T1w/T2w ratio | -0.06 | -3.65 | <.001* | 0.004* |
| Occupation - mean T1w/T2w ratio | -0.04 | -2.92 | 0.004* | 0.036* |
| Income - mean T1w/T2w ratio | -0.01 | -0.57 | 0.567 | 0.933 |

Supplementary Table 5. Associations between socioeconomic status (SES) and total cortical surface area, mean cortical thickness and mean cortical T1w/T2w ratio. The table depicts standardized beta coefficients, t statistics and uncorrected and corrected p values from linear models testing the relation between SES metrics and global cortical measures. * = Significant p values when using a threshold of *p* < .05

| Relationship | *β* | t | p | Corrected p |
| --- | --- | --- | --- | --- |
| Parental SES - total surface area | 0.04 | 1.73 | 0.085 | 0.255 |
| Education - total surface area | <-0.01 | -0.05 | 0.964 | 0.964 |
| Occupation - total surface area | 0.05 | 2.63 | 0.009* | 0.036* |
| Income - total surface area | 0.03 | 1.53 | 0.128 | 0.255 |

Supplementary Table 6. Association between socioeconomic status (SES) and total cortical surface area, corrected for estimated total intracranial volume. The table depicts standardized beta coefficients, t statistics and uncorrected and corrected p values from linear models testing the relation between SES metrics and total surface area. * = Significant p values when using a threshold of *p* < .05

*3.3 T1w/T2w ratio as a potential mediator of the parental SES- cognitive ability relations*

See Supplementary Table 7 and 8 and the main text for a presentation and discussion of findings regarding the effect of parental SES on specific cognitive abilities in youths, and non-significant mediatory effects of T1w/T2w ratio.

| ****Parental SES model comparison**** | ****z-statistic**** | ****p-value**** | ****Corrected p value**** |
| --- | --- | --- | --- |
| **Mean T1w/T2w-ratio -** mean cortical thickness | **-2.42** | **0.015*** | **0.046*** |
| **Mean T1w/T2w-ratio –** total surface area | **-0.8** | **0.426** | **0.426** |
| Mean cortical thickness ****–**** total surface area | **-0.8** | **0.426** | **0.426** |

**Supplementary Table 6. Effect size comparisons from the global MRI metric analyses and parental SES. The table shows the z-statistic and accompanying p values, from analyses comparing each of the models which assessed relations between parental SES and the global MRI metric, i.e. mean T1w/T2w ratio, total surface area and mean cortical thickness.**

| Cognitive ability | *β* | t | p | Corrected p |
| --- | --- | --- | --- | --- |
| Cognitive flexibility | -0.01 | -0.18 | 0.858 | 0.992 |
| Inhibitory control | 0 | -0.11 | 0.910 | 0.992 |
| Visual attention | 0 | -0.01 | 0.992 | 0.992 |
| Episodic memory | -0.01 | -0.36 | 0.718 | 0.992 |
| Processing speed | 0.08 | 2.09 | 0.037 | 0.225 |
| Oral reading skill | 0.14 | 5.06 | <.001* | <.001* |
| Working memory | 0.07 | 1.79 | 0.074 | 0.371 |
| Vocabulary knowledge | 0.18 | 5.75 | <.001* | <.001* |

Supplementary Table 7. Effect of SES on cognitive abilities. The table depicts standardized beta coefficients, t statistics and uncorrected and corrected p values from linear models testing the relation between SES metrics and specific cognitive abilities. * = Significant p values when using a threshold of *p* < .05

| Language ability | Hemispheric ROI | ACME | p | ADE | p | Total effect | p | Prop.  mediated | p |
| --- | --- | --- | --- | --- | --- | --- | --- | --- | --- |
| Reading | lh | 0.28 | 0.13 | 6.62 | <.001 | 6.90 | <.001 | 0.04 | 0.13 |
|  | rh | 0.28 | 0.12 | 6.62 | <.001 | 6.90 | <.001 | 0.04 | 0.12 |
| Vocabulary | lh | <.01 | 0.98 | 0.17 | <.001 | 0.17 | <.001 | <.01 | 0.98 |
|  | rh | <.01 | 0.78 | 0.17 | <.001 | 0.17 | <.001 | <.01 | 0.78 |

Supplementary Table 8. Mediation effect of T1w/T2w ratio on the relation between socioeconomic status (SES) and language abilities. The table shows coefficients, confidence intervals and p-values for the mediation model of T1w/T2w ratio on the relation between parental SES and reading and vocabulary. ACME= *“*average causal mediation effects”, **ADE= “average direct effect”, Prop.Mediated= “proportion mediated”.**

**References**

Brown, T. T., & Jernigan, T. L. (2012). Brain Development During the Preschool Years. *Neuropsychology Review*, *22*(4), 313–333. https://doi.org/10.1007/s11065-012-9214-1

Clogg, C. C., Petkova, E., & Haritou, A. (1995). Statistical Methods for Comparing Regression Coefficients Between Models. *American Journal of Sociology*, *100*(5), 1261–1293. JSTOR.

Esteban, O., Birman, D., Schaer, M., Koyejo, O. O., Poldrack, R. A., & Gorgolewski, K. J. (2017). MRIQC: Advancing the automatic prediction of image quality in MRI from unseen sites. *PLOS ONE*, *12*(9), e0184661. https://doi.org/10.1371/journal.pone.0184661

Glasser, M. F., Sotiropoulos, S. N., Wilson, J. A., Coalson, T. S., Fischl, B., Andersson, J. L., Xu, J., Jbabdi, S., Webster, M., Polimeni, J. R., Van Essen, D. C., & Jenkinson, M. (2013). The minimal preprocessing pipelines for the Human Connectome Project. *NeuroImage*, *80*, 105–124. https://doi.org/10.1016/j.neuroimage.2013.04.127

Jernigan, T. L., Brown, T. T., Hagler, D. J., Akshoomoff, N., Bartsch, H., Newman, E., Thompson, W. K., Bloss, C. S., Murray, S. S., Schork, N., Kennedy, D. N., Kuperman, J. M., McCabe, C., Chung, Y., Libiger, O., Maddox, M., Casey, B. J., Chang, L., Ernst, T. M., … Dale, A. M. (2016). The Pediatric Imaging, Neurocognition, and Genetics (PING) Data Repository. *NeuroImage*, *124*, 1149–1154. https://doi.org/10.1016/j.neuroimage.2015.04.057

Khundrakpam, B., Choudhury, S., Vainik, U., Al‐Sharif, N., Bhutani, N., Jeon, S., Gold, I., & Evans, A. (2020). Distinct influence of parental occupation on cortical thickness and surface area in children and adolescents: Relation to self‐esteem. *Human Brain Mapping*, *41*(18), 5097–5113. https://doi.org/10.1002/hbm.25169

Noble, K. G., Houston, S. M., Brito, N. H., Bartsch, H., Kan, E., Kuperman, J. M., Akshoomoff, N., Amaral, D. G., Bloss, C. S., Libiger, O., Schork, N. J., Murray, S. S., Casey, B. J., Chang, L., Ernst, T. M., Frazier, J. A., Gruen, J. R., Kennedy, D. N., Van Zijl, P., … Sowell, E. R. (2015). Family income, parental education and brain structure in children and adolescents. *Nature Neuroscience*, *18*(5), 773–778. https://doi.org/10.1038/nn.3983

Rakesh, D., & Whittle, S. (2021). Socioeconomic status and the developing brain – A systematic review of neuroimaging findings in youth. *Neuroscience & Biobehavioral Reviews*, *130*, 379–407. https://doi.org/10.1016/j.neubiorev.2021.08.027

White, N., Roddey, C., Shankaranarayanan, A., Han, E., Rettmann, D., Santos, J., Kuperman, J., & Dale, A. (2010). PROMO: Real-time prospective motion correction in MRI using image-based tracking. *Magnetic Resonance in Medicine*, *63*(1), 91–105. https://doi.org/10.1002/mrm.22176

Winkler, A. M., Ridgway, G. R., Webster, M. A., Smith, S. M., & Nichols, T. E. (2014). Permutation inference for the general linear model. *NeuroImage*, *92*, 381–397. https://doi.org/10.1016/j.neuroimage.2014.01.060

.
